# Complex Magnetic Fields: Harnessing the Electromagnetic Symphony for Possible Applications in Regenerative Medicine and Antifungal Properties

**DOI:** 10.1111/iwj.70679

**Published:** 2025-05-19

**Authors:** Muhammad Dawood Amjad, Lorenzo Marramiero, Tea Romasco, Alessandro Cipollina, Hamid Heydari Sheikh Hossein, Marco Mantarro, Adriano Piattelli, Stefania Fulle, Natalia Di Pietro, Rosa Mancinelli

**Affiliations:** ^1^ Department of Medical, Oral and Biotechnological Sciences “G. D'Annunzio” University of Chieti‐Pescara Chieti Italy; ^2^ Department of Neurosciences, Imaging and Clinical Sciences “G. D'Annunzio” University of Chieti‐Pescara Chieti Italy; ^3^ Center for Advanced Studies and Technology‐CAST, “G. D'Annunzio” University of Chieti‐Pescara Chieti Italy; ^4^ Independent Researcher Sciacca Italy; ^5^ Independent Researcher MFI Srl Rome Italy; ^6^ School of Dentistry Saint Camillus International University of Health and Medical Sciences Rome Italy; ^7^ Facultad de Medicina Universidad Católica San Antonio de Murcia (UCAM) Murcia Spain

**Keywords:** antifungal, complex magnetic fields (CMFs), oxidative stress, regenerative medicine, wound healing

## Abstract

Complex magnetic fields (CMFs) represent an emerging frontier in regenerative medicine, offering significant potential for innovative therapeutic strategies. This review examined both the theoretical foundations and practical applications of CMFs, focusing on their roles in tissue regeneration and antifungal activity. A comprehensive review of electronic databases (PubMed, Scopus, and Embase) identified seven pivotal studies on in vitro models concerning the CMF topic. Although the number of studies is limited, they collectively highlighted the promising therapeutic potential of CMFs in enhancing wound healing, reducing oxidative stress, and neuroinflammation in diabetic neuropathy, positively influencing mitochondrial function, modulating immune responses, promoting cellular communication, inhibiting the growth and adhesion of *Candida albicans* to medical surfaces, and enhancing dental pulp stem cell proliferation under inflammatory conditions. These findings suggested that CMFs may offer an eco‐sustainable approach, effectively targeting pathogens while preserving human cell integrity. While the current body of research is insightful, it remains in its early stages. To fully leverage the therapeutic potential of CMFs, more comprehensive studies are needed to refine their application and confirm their effectiveness across diverse clinical scenarios. This is essential for integrating CMFs into clinical practice, where they promise to revolutionise treatment approaches.

**Abbreviations**
AEF: alternating electromagnetic field
AIP: anti‐inflammatory program
AOSP: antioxidant support program
C: Coulomb
CE: European Conformity
CFU: colony‐forming unit
CLSM: confocal laser scanning microscopy
CMF: combined magnetic field
CMFs: complex magnetic fields
CT: computed tomography
DFU: diabetic foot ulcer
DM: diabetes mellitus
DN: diabetic neuropathy
DPSC: dental pulp stem cell
ECG: electrocardiogram
ECM: extracellular matrix
EF: electric field
ELF‐MF: extremely low‐frequency magnetic field
EMF: electromagnetic field
G: Gauss
hADMSC: human adipose‐derived mesenchymal stem cell
hGF: human gingival fibroblast
hMSC: human mesenchymal stem cell
Hz: Hertz
IL: interleukin
LPS: lipopolysaccharide
m/s: meter/second
MF: magnetic field
MMP: matrix metalloproteinase
MRI: magnetic resonance imaging
Non‐PSEMF: non‐pulsed sinusoidal electromagnetic field
PDLSC: periodontal ligament stem cell
PEMF: pulsed electromagnetic field
PRF: pulsed radiofrequency field
PRFE: pulsed radiofrequency electromagnetic
RF: radiofrequency field
RMF: rotating magnetic field
ROS: reactive oxygen species
SEM: scanning electron microscopy
SEMF: sinusoidal electromagnetic field
SMF: static magnetic field
T: Tesla
TES: transcranial electric stimulation
TMR: therapeutic magnetic resonance
TMS: transcranial magnetic stimulation

1

**Summary**

- Complex magnetic fields (CMFs) are a promising frontier in regenerative medicine, providing innovative strategies for tissue regeneration and antimicrobial activity.
- This review outlines the theory and practical uses of CMFs, especially in wound healing and antifungal treatment.
- CMFs improve wound healing, reduce oxidative stress, and mitigate neuroinflammation in diabetic neuropathy by enhancing mitochondrial function, adjusting immune responses, facilitating cell communication, preventing *C. albicans* growth on medical surfaces, and boosting dental pulp stem cell proliferation in inflammation.
- Limited studies show CMFs enhance wound healing, necessitating further research to refine their use and confirm benefits.
- CMFs offer an eco‐friendly method to target pathogens without harming human cells, providing a safer alternative to traditional therapies.
- If well‐developed, CMFs could improve patient outcomes and enable transformative advances in medical treatments.

## 2 Introduction

Magnetic fields (MFs) are a fundamental concept in physics, showcasing the intricate interactions among magnets, electric currents, and charged particles. This phenomenon has been rigorously investigated, yielding substantial insights and establishing itself as a pivotal element across various disciplines, including electromagnetism, technology, medicine, and astronomy [1].

According to the Lorentz force law, MFs can be described as vector fields that exert a force on moving charged particles. The force applied by these fields is orthogonal to both the velocity of the charged particle and the MF itself. Consequently, if the particle moves in parallel alignment with the MF, it will not experience any force. Conversely, if the charged particle traverses perpendicularly to the MF, it will encounter the maximum force. MFs are measured in units called Teslas (T) and may also be represented in Gauss (G), where 1 T corresponds to the force exerted by a MF on a charged particle of 1 C moving at a velocity of 1 m/s. For instance, the Earth's MF has an average intensity of approximately 0.5 G, which is equivalent to 50 μT [2].

A stationary charge generates an electrical field (EF) in the surrounding space, while the motion of the charge produces a MF. The interaction between the EF and MF results in the formation of an electromagnetic field (EMF). Consequently, as one of the fundamental forces of nature, the EMF is a physical field that arises when a charged entity is accelerated [3]. The EMF represents a type of physical field in which external forces generated by electrically charged objects are present. The EMF propagates through space in the form of electromagnetic waves (radiation), which consist of two interrelated components: MF and EF [4]. The potential difference between charge‐carrying bodies determines the strength of the EF, while the magnitude of the MF is proportional to the current flowing through a conductor [5]. For instance, charged particles that accumulate in the atmosphere following thunderstorms give rise to EFs, whereas the Earth possesses its own MF. In addition to natural sources, numerous artificial sources contribute to EMF generation, including televisions, computer screens, microwave ovens, mobile phones, and various medical devices such as X‐ray machines, computed tomography (CT) scanners, and magnetic resonance imaging (MRI) machines.

EMFs are typically classified according to their frequency, which refers to the number of waves that propagate per second and is measured in Hertz (Hz). They are primarily categorised into three distinct frequency ranges: extremely low‐frequency MFs (ELF‐MFs; up to 300 Hz), intermediate‐frequency fields (ranging from 300 Hz to 10 MHz), and radiofrequency fields (RFs; spanning from 10 MHz to 30 GHz) [3]. Moreover, EMFs can be further classified into additional categories including permanent MFs, low‐frequency sine waves, pulsed EMFs (PEMFs), pulsed RFs (PRFs), and transcranial magnetic/electric stimulation (TMS/TES). PEMFs generally consist of low‐frequency fields characterised by specific waveforms and amplitudes. The wide variety of commercially available PEMF devices presents challenges in comparing their physical and engineering properties, complicating the analysis of their biological and clinical effects. In contrast, PRFs operate at a frequency of 27.12 MHz and can function in two modes: continuous mode, which typically produces substantial heat, and pulsed (non‐thermal) mode, intended for the stimulation of soft tissues. Lastly, TMS involves the targeted activation of specific brain regions using brief magnetic pulses with intensities of up to 8 T [6, 7].

Unlike other electromedical devices used in instrumental physical therapy that primarily transfer energy in various forms, such as thermal, mechanical, electrical, and photonic, complex magnetic fields (CMFs) are distinguished by their ability to convey precise packets of information coherent with the cellular system (patterns) to biological tissues. This transfer of information is achieved by harnessing the beneficial properties of magnetic induction as a transport medium through complex multi‐frequency signals characterised by multiple harmonics, referred to as machine codes. CMFs represent magnetoelectric effect fields with a complex geometric configuration of multiple harmonics. The term ‘CMFs’ reflects the varying waveforms generated by the CMF device, which depend on specific energy requirements. These waveforms may be square, sinusoidal, impulsive, triangular, or trapezoidal, each exhibiting different rising and falling fronts characterised by varying steepness. This includes patented harmonic enrichment and specific control related to the modulation depth of the sorting signal. These MFs emerge from the necessity to simultaneously stimulate a broad range of cellular systems, facilitating a sequence of functions incorporating retroactive control. In this context, molecules exhibit distinct reactions based on their subtle chemical and diamagnetic properties. CMFs demonstrate their biological efficacy most effectively within the 1–250 Hz frequency range and at intensities ranging from 0.1 to 1 G. The selection of magnetic induction over alternative energy forms enables CMF systems to transfer bioactive therapeutic codes with minimal energy input. This characteristic has the potential to prevent tissue heating, circumvent the physical limitations associated with mechanical energy, mitigate the risk of intolerance to electrical currents, which could lead to tissue damage or burns, and transcend the penetration depth limitations of photonic energy [8].

The ability of CMFs to rapidly influence cellular and tissue repair mechanisms makes the investigation of their reparative capacity at these levels a particularly compelling area of research. Accordingly, this review aimed to summarise and critically assess the existing knowledge surrounding the application of CMFs, emphasising current applications as well as their prospective roles in regenerative medicine. This paper sought to elucidate contemporary studies on CMFs and underscore their potential as a non‐invasive therapeutic modality. Additionally, the breadth of available research on CMFs was examined, starting from a description of the state of the art in EMFs in regenerative medicine to the introduction of CMFs and their applications in tissue regeneration and antifungal activity, along with prospective research directions that could enable the full realisation of their therapeutic capabilities.

## 3 Methods

The present article is based on a literature search conducted through the PubMed, Scopus, and Embase databases, utilising the search terms ‘CMF’, ‘complex magnetic fields’, ‘extremely low‐frequency magnetic fields’, ‘CMF AND regeneration’, ‘CMF AND wound healing’, ‘CMF AND diabetes’, ‘CMF AND oxidative stress’, ‘CMF AND regenerative medicine’, ‘CMF AND antifungal activity’.

In total, 58 papers have been included in the final analytical synthesis and subsequently screened according to the authors' expertise to ensure alignment with the specific objectives of this review. Among them, only seven articles pertained to the CMF topic.

## 4 Results

### 4.1 State of the Art in EMFs in Regenerative Medicine

The historical relationship between EMFs and medicine represents a captivating evolution that has significantly influenced advancements in healthcare and medical technology over the centuries. This overview outlines critical milestones at the intersection of EMFs and medical practice. The connection between electricity and medicine can be traced back to the 17th century when physicians began experimenting with static electricity. They used devices such as the ‘electric machine’ to produce sparks and mild shocks for therapeutic applications, primarily aimed at treating nervous disorders and providing pain relief. In the 18th century, Franz Mesmer introduced the concept of ‘animal magnetism’, involving the use of magnets and hand gestures to address various health complaints. Although this practice was ultimately discredited as pseudoscience, it contributed to enhancing the understanding of the placebo effect and the psychological components of healing. The 19th century marked significant progress in applying EMFs for medical purposes, with pioneers such as Guillaume Duchenne and Nikola Tesla exploring the therapeutic potential of electricity and developing electrotherapy devices. Electrical currents were employed to stimulate muscles, alleviate pain, and treat neurological disorders [7]. Subsequently, in the early 20th century, Willem Einthoven unveiled the electrocardiogram (ECG or EKG), an innovative diagnostic tool for identifying cardiac conditions. This non‐invasive methodology records the heart's electrical activity, facilitating the detection of arrhythmias, heart attacks, and related issues [9]. The mid‐20th century witnessed a transformative milestone in medical technology with the advent of MRI. This technique utilises strong MFs and radio waves to generate detailed images of the body's internal structures, becoming essential for diagnosing a wide array of conditions affecting the brain, musculoskeletal system, and other areas [10]. During this period, electromagnetic therapy and stimulation techniques were employed to address various medical conditions. These therapies involved applying controlled EMFs to promote tissue repair, alleviate inflammation, and manage pain. Notable applications included PEMF therapy in orthopaedics for bone healing, wound care, and physiotherapy for pain management [11], as well as TMS for psychiatric disorders [12]. Furthermore, ionising radiation, a specific type of electromagnetic radiation, has been extensively used in cancer treatment. Particularly, high‐energy X‐rays and gamma rays were integral to radiotherapy and radiation oncology, effectively targeting and eliminating cancer cells [13].

Recent advancements in the study of EMFs have revealed promising developments concerning their potential to enhance the regenerative capabilities of living organisms. In this regard, regenerative medicine—an interdisciplinary field that integrates principles from engineering and life sciences—holds significant promise for rejuvenating damaged tissues and organs, thereby offering potential therapeutic solutions for various diseases and injuries. Regenerative medicine encompasses a diverse array of approaches, including the use of biomaterials and newly generated cells, both independently and in various combinations, to replace missing tissue. These strategies strive to restore the tissue's structural and functional integrity and facilitate the healing process [14]. Table 1 presents a comparison of different types of MFs used for bone tissue regeneration and details the models employed.

**TABLE 1:** Comparative analysis of the characteristics of magnetic fields (MFs) in bone tissue regeneration.

MF type	Model	Main results	Mechanism	References
SMF^a^	In vivo	Enhanced bone formation in human osteoblasts, periodontal ligament cells, and cementoblasts	SMFs activate the Wnt/β‐catenin pathway by enhancing Wnt protein expression and increasing β‐catenin accumulation. They also stimulate the MAPK (p38, JNK) and NF‐κB pathways, promoting osteoblastic and cementoblastic differentiation	[15]
SMF	In vivo	The exposure of MC3T3‐E1 cells to a MF of 500 nT and 0.2 T impaired cell differentiation. However, when exposed to a MF of 16 T, cell mineralization was accelerated, along with the presence of biochemical substances	16 T SMF upregulates TFR1 and FPN1, enhancing both iron uptake and export. This regulation aids in maintaining iron homeostasis, which supports osteoblast differentiation by ensuring adequate iron availability for metabolic and differentiation‐related processes	[16]
SMF	In vivo	Enhanced growth and development of human mesenchymal stem cells (hMSCs)^b^ using biochemical substances	Not detected	[17]
SMF	In vivo	Improved osseointegration of dental implants in humans	Not detected	[18]
SMF	In vitro	Enhanced bone healing in rats	Not detected	[19]
PEMF^c^	In vivo	Enhanced generation of new bone tissue by hMSCs derived from the supportive tissue of bone marrow, utilising biochemical substances	Increased expression of L‐type voltage‐gated Ca^2+^ channels and modulation of cytosolic free Ca^2+^ concentration. This elevation in Ca^2+^ upregulates osteogenic markers (ALP, osteocalcin, osteopontin) and enhances matrix mineralization, thereby facilitating bone formation	[20]
PEMF	In vivo	Unaffected proliferation of PDLSCs^d^; enhanced osteogenic differentiation when exposed to biochemicals	Not detected	[21]
PEMF	In vivo	Enhanced osteogenic differentiation of hADMSCs^e^ (using bioactive substances)	Not detected	[22]
SEMF^f^	In vitro	Enhanced osteogenesis, elevated metabolic activity, and reduced bone resorption in both metaphyseal and diaphyseal tissues of rat femurs	Not detected	[23]
PEMF	In vivo	Enhanced bone healing in a rat model of zygomatic bone defects utilising platelet‐rich plasma	Not detected	[24]
PEMF	In vitro	Improved bone density and structure, reduced decline in biomechanical strength in rats that had undergone ovariectomy	Promoted Wnt/LRP5/β‐catenin pathway signalling. Wnt proteins interact with the LRP5 receptor, stabilising β‐catenin in the cytoplasm, which then translocates to the nucleus to promote osteoblast differentiation and bone formation	[25]
PEMF	In vitro	Decreased bone loss due to disuse promotes skeletal anabolic activities in hindlimb‐unloaded rats	Enhanced Wnt/LRP5/β‐catenin pathway signalling. PEMFs partially preserve bone mass, microarchitecture, and strength by promoting bone formation through increased osteoblast activity, as shown by higher serum osteocalcin levels and improved bone formation surface	[26]
ELF‐MF^g^	In vitro	Reduced osteoporosis caused by spinal cord injury in rats	Not detected	[27]
RMF^h^	In vitro	Improved bone formation to repair an injured femoral head in experimental models of New Zealand rabbit	Not detected	[28]
Non‐PSEMF^i^	In vivo	Accelerated development of osteoblasts from hMSCs (with and without bioactive substances)	Not detected	[29]
AEF^j^	In vivo	Enhanced adult hMSCs differentiation into bone cells without utilising biochemical substances	Not detected	[30]
CMF^k^	In vitro	Increased bone growth at the interface of the bone and tendon in a rabbit model of partial patellectomy	Not detected	[31]

^a^ SMF: static magnetic field.

^b^ hMSCs: human mesenchymal stem cells.

^c^ PEMF: pulsed electromagnetic fields.

^d^ PDLSCs: periodontal ligament stem cells.

^e^ hADMSCs: human adipose‐derived mesenchymal stem cells.

^f^ SEMF: sinusoidal electromagnetic fields.

^g^ ELF‐MF: extremely low‐frequency magnetic field.

^h^ RMF: rotating magnetic fields.

^i^ Non‐PSEMF: non‐pulsed sinusoidal electromagnetic fields.

^j^ AEF: alternating electromagnetic fields.

^k^ CMF: combined magnetic field, a unique electromagnetic field that consists of a dynamic sinusoidal magnetic field and a magnetostatic field.

For example, static magnetic fields (SMFs) have been utilised in various in vivo and in vitro studies, demonstrating increased bone formation and cell differentiation, enhanced osseointegration of dental implants, and improved bone repair in rat models. The mechanisms behind these effects include the activation of key signalling pathways, such as Wnt/β‐catenin, JNK and p38 mitogen‐activated protein kinases (MAPK), as well as the nuclear factor kappa‐light‐chain‐enhancer of activated B cells (NF‐κB) pathways [15, 16, 17, 18, 19]. Additionally, PEMFs have been shown to promote new bone tissue formation, facilitate osteogenic differentiation, and assist in bone repair. The mechanisms involved in these processes encompass the modulation of calcium channels and the Wnt signalling pathway. While other studies have reported varying degrees of effectiveness in enhancing bone formation, metabolic activity, and reducing bone resorption, many mechanisms by which these effects occur remain to be clarified [20, 21, 22, 23, 24, 25, 26].

Table 2 provides a summary of clinical studies examining the effects of various types of MFs on diabetic foot ulcers (DFUs).

**TABLE 2:** Clinical perspectives on the impact of MFs on diabetic foot ulcers (DFUs).

MF type	Exposure duration	Patients enrolled	Treatment group	Main findings	References
PEMF	60 min/session, 14 sessions over three weeks	13 patients diagnosed with type 2 diabetes who have experienced poor healing of an ulcer in the past four weeks	Two groups, one receiving PEMF treatment and the other not	The PEMF group experienced an 18% decrease in wound size and a significant increase in both cutaneous capillary blood velocity and capillary diameter	[32]
ELF‐MF	Forearm: 2 h/day, twice a week; Thorax: 25 min/day, twice a week	26 patients with diabetes who have non‐responsive DFUs	All received ELF‐MFs	No negative consequences, and the ulcers did not reemerge at the original sites	[33]
PRFE^a^	Six–eight h/day for six consecutive weeks	Four individuals with DFUs that have lasted for over three months	All received PRFE	Within a week, the size of ulcers in diabetic patients that had persisted for over three months decreased. After three weeks of treatment, two patients fully healed their ulcers	[34]
PEMF	Two weeks in a row, 20 min each, twice daily	A group of 20 individuals diagnosed with diabetes mellitus for over five years who are experiencing DFUs	Two groups: one with the application of TMR^b^ and one without it	Improved healing speed and shorter healing time at six months, along with faster granulation tissue formation at three months, without any significant negative effects	[35]
PEMF	Two weeks in a row	40 patients diagnosed with type 2 diabetes for at least five years developing a distal neuropathic ulcer on their foot that has persisted for more than six weeks and is larger than 1 cm^2^	Two groups: one using TMR and one without using it	Enhanced healing rate, accelerated tissue regeneration, improved formation of healthy granulation tissue, better differentiation of keratinocytes, increased collagen fibre deposition, higher levels of collagen, integrin‐α1, integrin‐β3, MMPs^c^, cytoskeletal proteins, interleukins, and decreased expression of pro‐inflammatory cytokines	[36]

^a^ PRFE: pulsed radiofrequency electromagnetic.

^b^ TMR: therapeutic magnetic resonance.

^c^ MMPs: matrix metalloproteinases.

PEMFs have shown significant improvements in wound management, including an 18% reduction in wound size, increased blood velocity, enhanced capillary diameter, accelerated healing processes, and tissue regeneration, all achieved without adverse effects [32]. ELF‐MFs have demonstrated no negative consequences or recurrence of ulcers [33]. Furthermore, pulsed radiofrequency electromagnetic fields (PRFEs) have been observed to significantly reduce ulcer size within one week and achieve complete healing in some cases within three weeks [34]. Additional studies have emphasised the effectiveness of these therapeutic approaches, particularly PEMFs, in promoting granulation tissue formation, collagen fibre deposition, keratinocyte differentiation, and in reducing levels of pro‐inflammatory cytokines, which are vital for the healing of ulcers [35, 36].

### 4.2 CMFs

The operational principle of CMFs relies on complex, multi‐harmonic MFs to carry specific biological information, characterised by four fundamental parameters: signal frequency, induction intensity, action time, and waveform.

**FIGURE 1:**
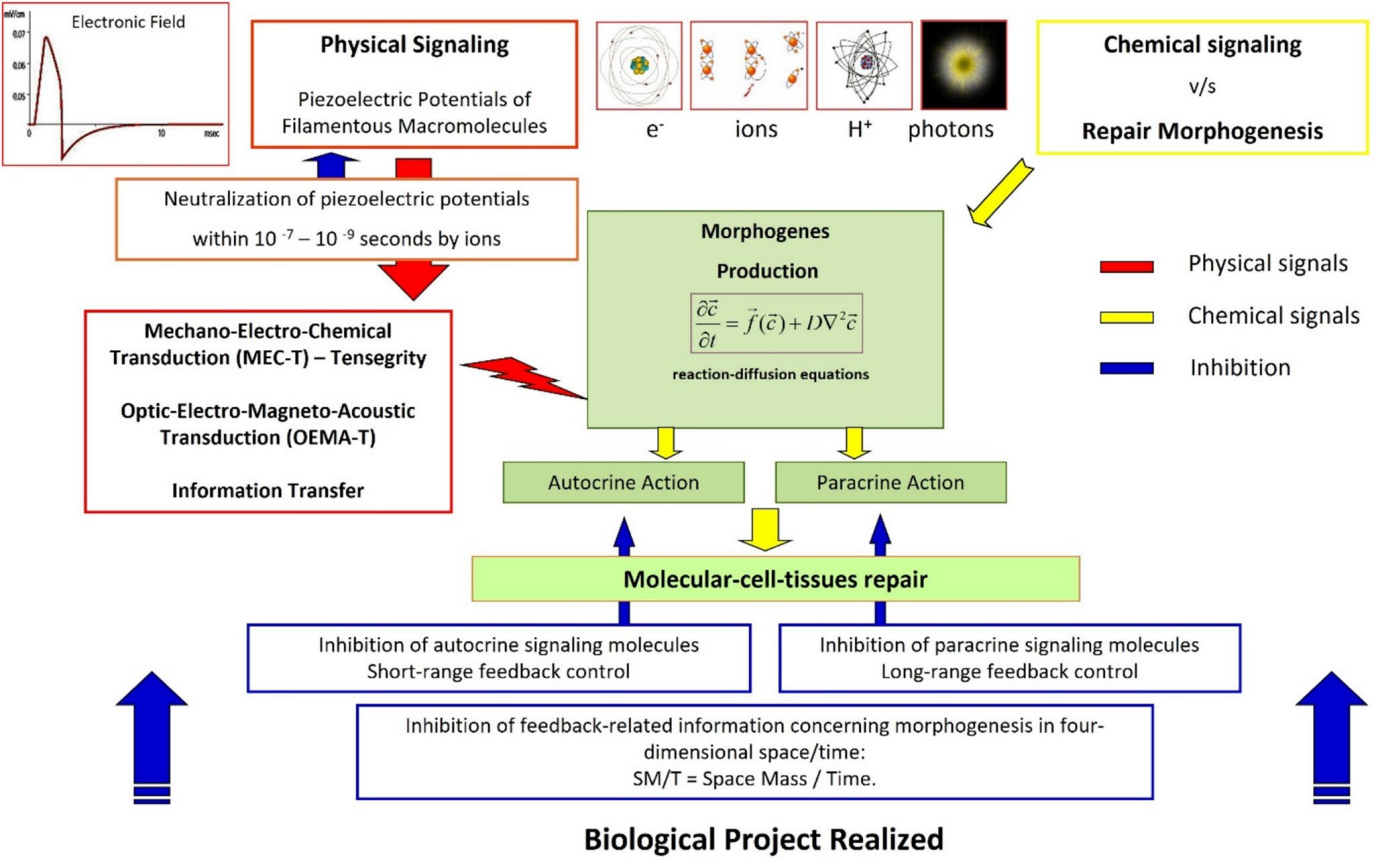
Mechanism of action for complex magnetic fields (CMFs): an examination of the informational pathways involved in repair morphogenesis and an analysis of both short‐range and long‐range feedback control within a biophysical model.

Integrating these four parameters, along with their sequential arrangement and physiological alignment within designated programs, allows for the synchronised activation of biological processes that promote healing and/or rebalancing of the organism. These parameters establish precise cellular communication codes (machine codes), facilitating the simultaneous stimulation of various cell types, depending on the biological objectives to be achieved. The therapeutic effect primarily arises from the ability of the machine codes to interact with diverse biological tissues, while the MF itself acts solely as a transporter of these codes (Figure 1) [8].

**FIGURE 2:**
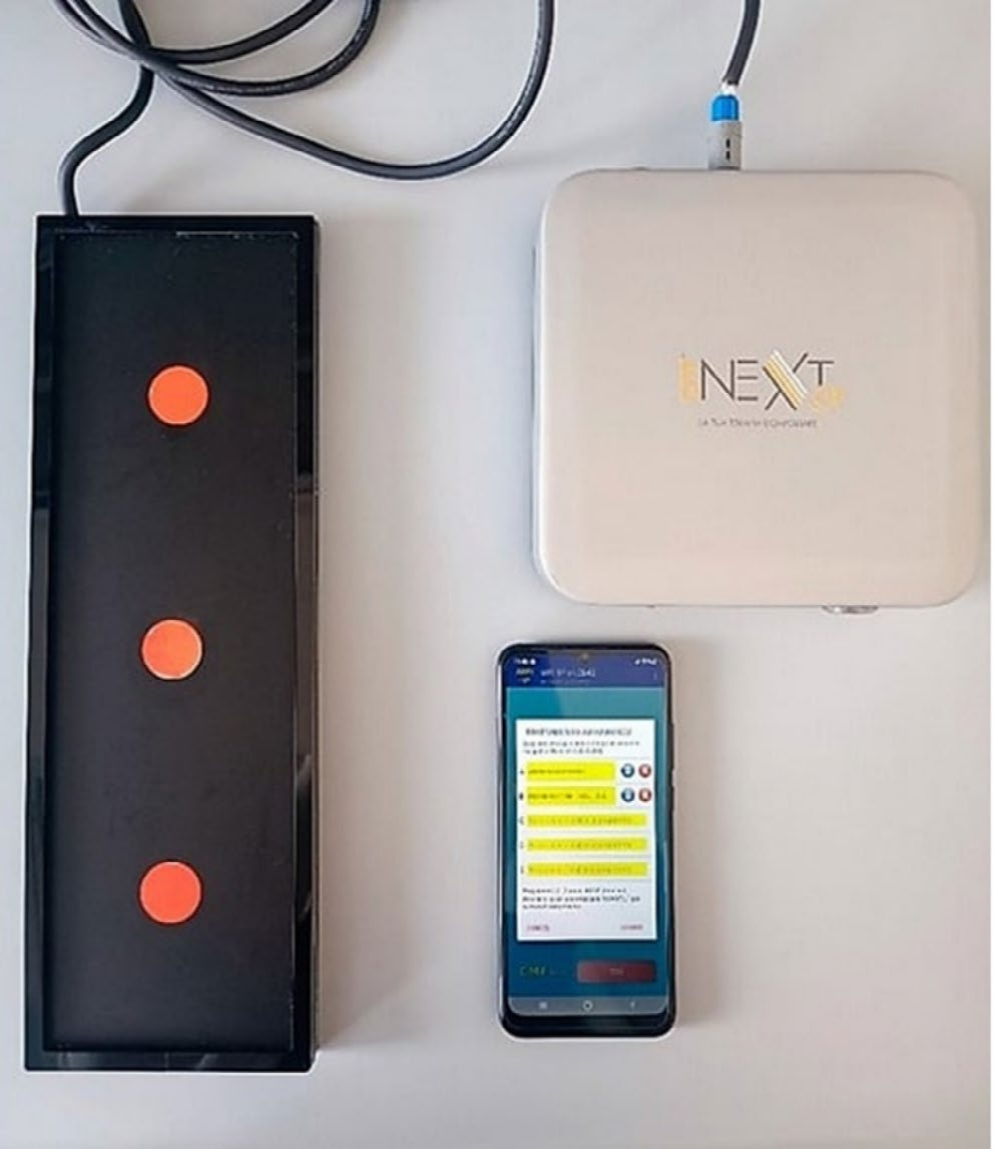
The CMFs device (Next SX version; Medicina Fisica Integrata M.F.I., Rome, Italy) is equipped with a CMF generator, a support structure for cell plates and flasks, and a smartphone that runs the MFI‐1P software (version 5.0; Medicina Fisica Integrata M.F.I., Rome, Italy), which is used to program the CMF generator. *Source:* This figure is derived from the research conducted by Gallorini et al. [37].

One of the fundamental characteristics of the programs offered by CMF devices (CMF and CMF NEXT; Medicina Fisica Integrata M.F.I., Rome, Italy) is their alignment with the natural physiology of healing processes (Figure 2). More specifically, these programs assist in rebalancing the body.

The pre‐programmed, tissue‐specific therapeutic protocols generally encompass multiple stages, which are carefully arranged in a specific sequence to align with the desired biological process. The types of signals emitted by the devices within the CMF systems are highly effective from a therapeutic perspective while ensuring exceptional safety during operation, resulting in virtually no side effects. The emission frequency remains extremely low, situated within the ELF range, with induction intensity maintained in the μT range. Moreover, therapy sessions typically do not exceed 30 min per program. The application of CMFs fosters a genuine communication process with the cellular system from a physical standpoint, thus enabling the effective treatment of diseases that may challenge traditional medicine. In addition to established methods of regenerative medicine, CMFs offer a non‐invasive and potentially transformative alternative. Although current research in this field is limited, it is hypothesized that these therapies may produce both immediate and long‐term effects through their specific mechanisms of action. These include the reduction of reactive oxygen species (ROS), modulation of ionic strength, resonance of calcium ions, effects on membrane channels, acceleration of mitosis and meiosis, facilitation of ligand–receptor interactions (Stark effect on macromolecules), action on transmembrane proteins, enhancement of enzyme kinetics, influence on the gene expression of growth factors, increased cytoprotection, and the demonstration of anti‐inflammatory and antifungal properties, all of which contribute to promoting cell proliferation [38, 39, 40, 41, 42, 43, 44, 45, 46, 47, 48].

### 4.3 Current Applications of CMFs

**FIGURE 3:**
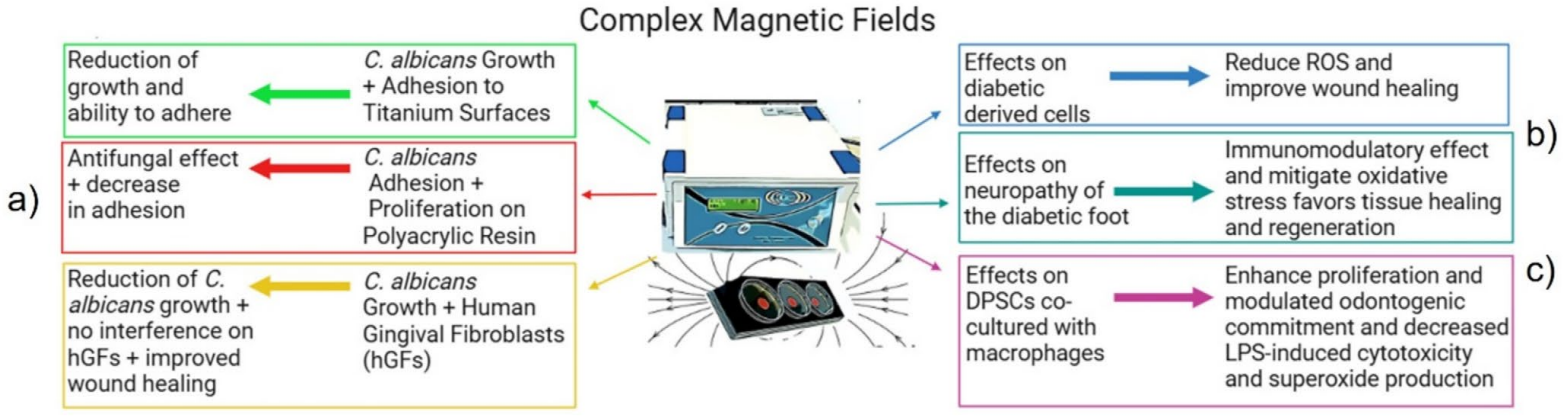
Recent advancements in the applications of CMFs. (a) CMFs applied to *C. albicans* inhibit its growth and adhesion, demonstrating antifungal properties; (b) CMFs reduce reactive oxygen species (ROS) and enhance wound healing in cells derived from diabetic patients, while also alleviating neuropathic complications associated with diabetic foot ulcers (DFUs) (c) CMFs promote proliferation and decrease cytotoxicity and superoxide production in dental pulp stem cells (DPSCs) when co‐cultured with macrophages. This figure was created using BioRender.com.

Current applications of CMFs in treating diabetic neuropathy, promoting wound healing, and exhibiting antifungal activity incorporate innovative methodologies aimed at accelerating tissue regeneration and combating fungal infections through regulated magnetic stimulation. A notable reduction in antimicrobial activity and adherence has been observed. Furthermore, CMFs modulate oxidative stress and enhance regeneration processes. They also promote the proliferation of dental pulp stem cells (DPSCs) under pro‐inflammatory conditions and facilitate odontogenic commitment (Figure 3).

#### 4.3.1 CMFs and Their Role in Enhancing Wound Healing Process

The wound healing process can be significantly hindered by various conditions, including ischemia, infection, and skin injury. A common pathway associated with these processes involves the role of ROS in wound healing. Diabetic foot disease, the leading cause of morbidity among individuals with diabetes, exhibits both functional and structural changes, particularly the presence of ulcers often accompanied by osteomyelitis or gangrene, arising from chronic inflammation and endothelial dysfunction [49, 50]. Due to a hyperglycaemic state, endothelial cells shift from using nitric oxide to metabolising glucose, which leads to impaired vasodilation [51]. This physiological change induces an imbalance between free radicals and antioxidants, resulting in the production of excessive ROS [52]. DFUs are among the most common manifestations of diabetes mellitus (DM), contributing to disability and an increased risk of mortality. A recent study conducted by Zanotti et al. [38] investigated the effects of CMFs on ROS production and wound healing characteristics in fibroblast and monocyte cultures derived from diabetic patients. The findings confirmed the complete compatibility of the treatment with living tissues and the absence of adverse impacts on cellular function, in accordance with ISO 10993‐5:2009 standard guidelines. Additionally, the results indicated that CMF treatment significantly reduced ROS production, promoted the anti‐inflammatory M2 phenotype in macrophages through the activation of miRNA 5591, decreased levels of inflammatory cytokines such as Interleukin‐1 (IL‐1) and Interleukin‐6 (IL‐6), and increased levels of anti‐inflammatory cytokines including Interleukin‐10 (IL‐10) and Interleukin‐12 (IL‐12). Furthermore, the treatment enhanced the expression of markers associated with improved wound healing, such as type I collagen and integrins. These findings suggest that CMFs facilitate a reduction in ROS formation within an inflammatory environment. Given the established role of ROS in various aspects of wound healing—such as macrophage polarisation, bacterial elimination, and extracellular matrix (ECM) cross‐linking—mitigating excessive ROS levels may substantially enhance tissue healing and regeneration.

#### 4.3.2 CMFs and Their Effects on Mitochondrial Function in Diabetic Neuropathy

Diabetic neuropathy (DN) affects 50%–60% of patients with DM, leading to progressive nerve damage that results in atrophied nerves and irreversible disability. Clinically, this condition presents a range of symptoms, from numbness to persistent pain [53]. Managing DN‐related pain poses significant challenges due to inadequate diagnostic criteria and limited treatment options. The study conducted by Chianese et al. [54] investigated the effects of CMFs on glial cells derived from the in vitro differentiation of mesenchymal stem cells (MSCs), utilising this model to explore diabetic foot neuropathy. This research employed the antioxidant support program (AOSP) and the anti‐inflammatory program (AIP) to evaluate their effects on mitochondrial activity and miRNA expression. The cells received daily treatments over a period of up to five days. Initial assessments included a thiazolyl blue tetrazolium bromide (MTT) assay to correlate cell viability rates with mitochondrial function. The findings showed no pathogenic changes across all cell types, including those derived from MSC differentiation. The safety of these programs was confirmed through Ames and hemocompatibility testing in accordance with ISO 12000 guidelines. The biological pathways activated by the programs were examined through the analysis of miRNAs, which serve as primary biological entities responsible for the activation or inhibition of various metabolic pathways. Furthermore, the miRNA investigation revealed that CMFs may positively influence cell cycle regulation, as indicated by the overexpression of miRNAs from the 121, 127, and 142 families, which are associated with mitochondrial function and cell cycle control. Additionally, CMF therapy has demonstrated effectiveness in reducing oxidative stress by addressing ROS, thereby fostering an optimal environment for tissue repair. Moreover, CMF therapy has been shown to affect several components of the immune system, including immune cell proliferation, cytokine production, and inflammatory responses. Oxidative stress, marked by an imbalance between ROS production and the body's antioxidant defences, can impede tissue healing and regeneration. CMF treatment has proven to provide antioxidative support by reducing ROS levels and enhancing antioxidant enzyme activity. By reducing oxidative stress, CMF treatment promotes cellular proliferation, differentiation, and tissue remodelling, facilitating the regenerative process. This research may hold significant implications for preventing neuropathic complications in diabetic foot conditions.

#### 4.3.3 CMFs' Antifungal Activity

Antimicrobial resistance represents one of the most significant challenges in contemporary healthcare [55]. This concerning global phenomenon includes a diverse array of microorganisms, notably human pathogenic yeasts such as *Candida* spp. [56]. *C. albicans* stands out as the most potent yeast found in the oral cavity [57]. This yeast can adhere to oral epithelium and denture surfaces, proliferate, form biofilms, and subsequently disseminate throughout the oral cavity.

A study conducted by D'Ercole et al. [47] investigated the effects of several CMF programs on *C. albicans* in both planktonic and sessile stages, as well as on human gingival fibroblasts (hGFs). The findings demonstrated the efficacy of CMFs in significantly affecting the viability, virulence, and adhesion properties of *C. albicans* to titanium surfaces. This was achieved through the application of oxidative stress, oxidative stress/antibacterial, antibacterial, and antibacterial/oxidative stress programs of the CMF device. Notably, the cultivability and viability of *C. albicans* were significantly reduced following exposure to CMFs. This finding marked a crucial advancement in addressing the issue of drug resistance in *C. albicans* and suggested novel intervention strategies. Furthermore, the application of CMFs inhibited *C. albicans* 's ability to adhere to titanium implant surfaces and reduced hyphal production. Importantly, these interventions did not adversely affect the viability of hGFs. This study indicated that CMFs may serve as effective measures for preventing and treating yeast biofilm infections.

Denture wearers have an increased likelihood of developing halitosis, oral candidiasis, and various forms of stomatitis due to heightened local and systemic risk factors [58, 59]. Polyacrylic resin dentures present localised risk factors for several reasons. *C. albicans* tends to colonise the surfaces of these dentures. Furthermore, the pressure and friction exerted by the prosthesis can potentially damage the oral mucosa, facilitating the penetration of yeasts into the oral epithelium. In a separate study conducted by Petrini et al. [48], the researchers evaluated the impact of various sessions of the antibacterial protocol of CMFs on planktonic *C. albicans* , as well as on its ability to adhere and proliferate on acrylic resin materials. Four distinct test groups were analysed: one group treated solely with the initial program of the antibacterial protocol; another subjected to the first five programs of the protocol; a third that underwent one complete session of the antibacterial treatment; and a fourth that received two complete sessions of the antibacterial protocol. The findings revealed that CMFs resulted in a significant reduction in colony‐forming units (CFUs) of *C. albicans* . Additionally, scanning electron microscopy (SEM) observations, combined with CFU analysis, demonstrated that CMFs effectively diminished both the adhesion and growth of *C. albicans* on resin discs. The CMFs exhibited notable antifungal properties, leading to reduced adherence of *C. albicans* on polyacrylic resin surfaces.

The concerning issue of antibiotic resistance has prompted the pursuit of innovative eco‐sustainable strategies to address diseases linked to resistant microorganisms. A study conducted by Di Lodovico et al. [60] demonstrated that CMFs offer an eco‐sustainable solution to mitigate infections caused by resistant microorganisms, presenting a non‐invasive, chemical‐free, and energy‐efficient approach to suppress *C. albicans* growth without adversely affecting hGFs. The primary objective of their study was to assess the efficacy of two CMF programs—stress and antibacterial—against clinically antifungal‐resistant *C. albicans* while also evaluating their effects on hGFs. The study noted that CMFs significantly reduced the biomass of resistant *C. albicans* , with the stress program exhibiting particularly remarkable effects. Confocal laser scanning microscopy (CLSM) and SEM images illustrated a significant anti‐biofilm effect attributed to the STRESS program, highlighting CMFs' capacity to disaggregate the *C. albicans* biofilm and exert lethal effects. The CMFs disrupted the typical fluidity of *C. albicans* , increasing the stiffness of its membrane, as evidenced by elevated excitation generalised polarisation (GPexc) values compared to control samples. This alteration indicates an imbalance among microorganisms, making them more susceptible to therapeutic interventions. Furthermore, the study found no detrimental effects of CMFs on hGF viability, even with heightened ROS generation observed through the antibacterial program. In wound healing assays, the stress program demonstrated the most significant impact on the migration rate of hGFs, while the antibacterial program significantly enhanced collagen production compared to both the control and stress programs. Overall, CMFs exhibited significant anti‐virulence activity against *C. albicans* , with no cytotoxic effects and improved migration properties in hGFs. The antifungal effects against *C. albicans* , combined with the absence of biological effects on hGFs, may be attributed to the differences in elastic modulus between eukaryotic cells and both bacterial and fungal cells.

The findings from this research on *C. albicans* suggest that CMFs hold promise as an innovative, eco‐friendly method for addressing resistant yeast biofilm infections, unlike conventional antifungal treatments that may exacerbate chemical pollution and resistance. CMFs function through physical mechanisms, thereby minimising environmental impact and the risk of drug resistance. Additionally, their low energy requirements and absence of toxic residues position them as a sustainable alternative for biomedical applications. The biocompatibility and selective action of CMFs towards microorganisms further underscore their potential in infection control while maintaining cellular health and environmental safety.

#### 4.3.4 Modulation of Dental Pulp Stem Cells (DPSCs) by CMFs in Inflammatory Co‐Cultures

Despite the growing awareness and emphasis on oral health, inflammatory dental diseases continue to pose significant clinical challenges, with their underlying causes and progression inadequately understood. During the development of dental caries, bacteria infiltrate the tooth pulp, leading to pulpitis. To prevent pulp necrosis, promoting tissue repair through the recruitment of immune cells, such as macrophages, is crucial. These cells can secrete signalling molecules that positively influence the pulp microenvironment and facilitate the recruitment of DPSCs to the site of damage. A study conducted by Gallorini et al. [37] examined the potential of CMFs to restore redox balance in a co‐culture model of DPSCs and inflamed macrophages, simulating an inflammatory condition similar to pulpitis. Their findings indicated that CMFs enhanced the proliferation of DPSCs under pro‐inflammatory conditions. Furthermore, CMF treatment resulted in a decrease in lipopolysaccharide (LPS)‐induced cytotoxicity and superoxide production. The modulation of key markers, including CD90, CD105, CD73, and CD29—crucial for the odontogenic commitment of DPSCs—in conjunction with CD14, which is associated with M1 polarisation of macrophages, was positively influenced following a dual treatment approach with CMFs. The researchers concluded that CMFs, by modulating both the odontogenic commitment and the anti‐inflammatory response of DPSCs, may serve as a promising therapeutic strategy for addressing pulpitis and, more broadly, dental inflammatory diseases.

## 5 Discussion

CMFs exert their biological effects through a combination of biophysical and biochemical mechanisms, as demonstrated by recent studies investigating their impact on microbial growth, inflammation, and cellular function [37, 38, 47, 48, 58, 60]. In the context of *C. albicans* , CMFs have been shown to disrupt its adhesion and proliferation on various surfaces, including titanium and polyacrylic resin. This disruption occurs by interfering with ionic fluxes, membrane potentials, oxidative stress pathways, biofilm formation, and metabolic activity. Notably, this antimicrobial action did not adversely affect hGFs, indicating the selective nature of CMFs in disrupting microbial cell homeostasis while preserving host tissues [47, 48].

Furthermore, in inflammatory models, CMFs appear to modulate immune responses. Studies involving DPSCs co‐cultured with macrophages have shown alterations in their immunophenotype and differentiation potential upon exposure to CMFs [37]. This implies that CMFs may influence cell signalling pathways, potentially by modifying ROS dynamics and mitochondrial function. Additional research on diabetic‐derived cells and neuroinflammation corroborated these findings, suggesting that CMFs have the capacity to mitigate oxidative stress and neuroinflammation, likely through interactions with mitochondrial function and redox balance modulation [38, 54].

Collectively, these findings indicate that CMFs orchestrate a biophysical modulation of both cellular and microbial processes, presenting a promising strategy for antifungal therapies, inflammation management, and tissue regeneration. However, the existing body of research on CMFs predominantly consists of preliminary investigations, often limited to small‐scale studies. This limitation subsequently constrains the generalisability of the findings and hinders the ability to draw robust conclusions regarding the efficacy and safety of CMFs in broader clinical contexts. While there is a foundational understanding of the potential impacts of CMFs on biological processes—such as the reduction of ROS and the modulation of calcium ion resonance—the precise molecular mechanisms underlying these effects remain inadequately elucidated. This gap in knowledge obstructs the formulation of optimised protocols for clinical applications. Moreover, the absence of standardised parameters—such as frequency, intensity, and duration of exposure—complicates the comparison of results across diverse studies. Such variability may lead to inconsistent outcomes and presents challenges to the reproducibility of findings.

A concerted effort to investigate the biological mechanisms of CMFs will facilitate the development of targeted therapeutic strategies. Gaining insight into how CMFs interact at the cellular and molecular levels is essential for creating more effective and individualised treatment protocols. Additionally, establishing standardised protocols for implementing CMFs, including optimal frequencies, intensities, and exposure durations, will enhance the consistency and replicability of results across various studies. Encouraging interdisciplinary collaboration will also promote the evolution of more advanced and effective CMF devices. Such collaborative approaches can accelerate the translation of basic research findings into clinical applications. By addressing these critical areas, the potential of CMFs in regenerative medicine and other therapeutic applications can be more comprehensively realised, paving the way for innovative and effective healthcare solutions.

Despite certain limitations, CMFs are considered harmless due to the low intensities and frequencies used in their application. Additionally, the CMF device has already obtained certification according to European Conformity (CE; UNI EN ISO 9001 and UNI EN 13485) and holds patent protection. Besides their demonstrated effectiveness in treating various conditions in in vitro models, a significant advantage of CMF technology is its potential to support the body in regaining and maintaining natural physiological balance. Consequently, it can be hypothesized that periodic treatment using various CMF protocols may contribute to the prevention of inflammatory and microbial diseases.

## 6 Conclusions

In conclusion, the investigation of CMFs revealed a dynamic and multifaceted area within the disciplines of physics and medicine, demonstrating the significant potential of these fields across various applications. The review of existing literature and studies highlighted the importance of CMFs in advancing our understanding and enhancing treatment methodologies for a wide array of medical conditions. The evidence supporting the effectiveness of CMFs in promoting wound healing, reducing oxidative stress, and exhibiting antifungal properties provided a solid foundation for future research and clinical applications. Furthermore, the therapeutic impact of CMFs on DFUs and DN indicated their potential in addressing complications related to chronic health issues. Research suggests that CMFs can decrease ROS, modulate immune responses, and facilitate cellular communication, thereby improving healing outcomes. The ability of CMFs to inhibit the planktonic growth of *C. albicans* and prevent its adhesion to titanium and polyacrylic resin surfaces showcased a novel, eco‐sustainable approach to infection management, especially in the context of dental and medical implants. Additionally, CMFs have been shown to promote the proliferation of DPSCs under pro‐inflammatory conditions. Notably, treatment with CMFs also decreased LPS‐induced cytotoxicity and superoxide production. These findings not only illustrated the potential of CMFs in managing microbial colonisation without negatively affecting hGFs but also suggested broader implications for maintaining oral hygiene and enhancing the longevity of implantable devices.

Overall, this review emphasised the promising capabilities of CMFs and advocated for ongoing research to fully realise their therapeutic potential, ultimately contributing to improved patient outcomes and the advancement of medical science. CMFs offer a wide range of potential applications across medicine, biotechnology, and industry. In healthcare, they may serve as a promising alternative to traditional antifungal and antimicrobial treatments by inhibiting the growth and adhesion of pathogens like *C. albicans* without harming human cells. This makes them particularly valuable for preventing infections in dental and orthopaedic implants, as well as medical prosthetics. Their ability to enhance cell proliferation, reduce inflammation, and promote wound healing suggests applications in regenerative medicine, diabetic ulcer treatment, and post‐surgical recovery. Additionally, CMFs demonstrate neuroprotective potential by improving mitochondrial function and reducing oxidative stress, which could assist in managing conditions such as neuroinflammation, DN, and neurodegenerative diseases like Alzheimer's and Parkinson's. Their immunomodulatory effects may also benefit individuals with autoimmune disorders, arthritis, and chronic inflammatory conditions. In cardiovascular health, they may aid in improving circulation, vascular healing, and preventing blood clots. In agriculture, CMFs have the potential to prevent fungal contamination in crops and stored food, offering an eco‐friendly alternative to chemical fungicides. As research progresses, CMFs are positioned to become a transformative, non‐invasive, and sustainable technology across multiple fields.

## Ethics Statement

The authors have nothing to report.

## Consent

The authors have nothing to report.

## Conflicts of Interest

The authors declare no conflicts of interest.

## Data Availability

Data sharing is not applicable to this article as no new data were created or analyzed in this study.
